# Prevalence and predictors of perceived disrespectful maternity care in postpartum Iranian women: a cross-sectional study

**DOI:** 10.1186/s12884-020-03124-2

**Published:** 2020-08-14

**Authors:** Khadije Hajizadeh, Maryam Vaezi, Shahla Meedya, Sakineh Mohammad Alizadeh Charandabi, Mojgan Mirghafourvand

**Affiliations:** 1 Midwifery Department, Tabriz University of Medical sciences, Tabriz, Iran; 2 Department of obstetrics and gynecology, Alzahra teaching hospital, Tabriz University of Medical Sciences, Tabriz, Iran; 3 School of Nursing, Faculty of Science, Medicine and Health, University of Wollongong, Wollongong, Australia; 4 Department of Midwifery, Faculty of Nursing and Midwifery, Tabriz University of Medical Sciences, Tabriz, Iran; 5 Social Determinants of Health Research Center, Tabriz University of Medical Sciences, Tabriz, Iran

**Keywords:** Disrespectful maternity care, Abuse, Prevalence, Predictor, Iran

## Abstract

**Background:**

Disrespectful maternity care is a key impediment to achieving a good quality care. Identifying predicting factors can be used in mitigating any potential risk in for disrespect and abuse in maternity care. The present study was conducted to determine prevalence and predictors of perceived disrespectful maternity care among Iranian women.

**Methods:**

A cross-sectional study was conducted in three public and three private hospitals in the city of Tabriz involving 334 postpartum women. Tools included socio-demographic, pregnancy, labour and birth characteristics questionnaires, and disrespect and abuse scales. Data were collected in 6 to 18 h after birth. Multivariate logistic regression was used to determine predictors of disrespectful maternity care.

**Results:**

A majority of the women (253; 75.7%) reported one or several types of perceived disrespectful maternity care. The most frequent types related to not allowing women to choose labour positions (142; 44.3%) and not allowing them to move during labour (148; 42.5%). Nighttime childbirth (aOR 3.07; 95% CI 1.61 to 5.88) increased the likelihood of perceived disrespectful maternity care. However, presence of spouses to accompany their wives in waiting rooms (aOR 0.32; 95% CI 0.11 to 0.88), the attendance of private physicians (aOR 0.05; 95% CI 0.02 to 0.12), and midwives (aOR 0.22; 95% CI 0.11 to 0.45) decreased the likelihood of perceived disrespectful maternity care.

**Conclusion:**

The results showed high levels of perceived disrespectful maternity care in postpartum women. Therefore, appropriate interventions, such as encouraging spouses’ presence, increasing the number of night shift staff, and training obstetric residents and midwives by holding ethics classes, with particular emphasis on empathy with patients.

## Background

The World Health Organization (WHO) has issued a statement on immediate attention for disrespect and abuse (D&A) during childbirth, which emphasized the importance of respectful maternity care (RMC) and women’s rights during pregnancy and childbirth [1]. RMC is an approach to care which emphasizes the fundamental rights of women, newborns, and families [2]. D&A is defined as abuse, violence, and inhuman or humiliating care that can occur both individually (by health care workers) and structurally (by systematic environmental defects) [3]. Analysis by Bowser & Hill divides D&A during childbirth into seven types: physical abuse, discrimination, non-consensual care, non-dignified care, non-confidential care, abandonment of care, and detention in facilities [4]. Verbal abuse and embarrassment, performing medical interventions such as non-emergency caesarean section, episiotomy, tubectomy, blood transfusion and hysterectomy without permission or informed consent; violation of women’ privacy, refusing pain relief when requested, not allowing presence of spouses and family members during childbirth, not allowing women to decide positions during childbirth, separation of mother and child have also been reported [4, 5].

Disrespectful care towards women during labour and childbirth has increased over the recent decade [6] and studies reported different prevalence of D&A in different settings such as 20% in Kenya [7] and 98% in Nigeria [8]. A study in Tanzania reported a prevalence of D&A of 15% immediately after birth and about 70% in follow-up interviews [9]. A cross-sectional study in five southern and eastern African countries reported abandoning and neglecting pregnant women during childbirth as the most common type of D&A [10]. Meanwhile, a study in Kenya and Nigeria reported non-dignified and non-consensual care, and physical abuse as the most common types of D&A [7, 8].

There is evidence that women subjected to D&A during childbirth may not seek vaginal birth [11, 12]. A study in six European countries showed that D&A during childbirth increases fear of childbirth and there is a significant relationship between this and the desire to opt for caesarean section [11].

Despite exciting D&A in some maternity care facilities, no single factor have been explained why some staff commit D&A towards women in labour, or why it is more prevalent in some regions [13]. D&A can result from complex interpersonal interactions, sociocultural and health system factors, and understanding these factors can be important for prevention [12]. In a qualitative study in Ethiopia, health care workers reported most types of D&A as unintentional, due to health system weaknesses and shortages of equipment [6]. Young age, being unmarried, poor socioeconomic status, women with HIV, higher parity, and childbirth complications have been related with higher levels of disrespectful maternity care [1, 7].

Considering that there is no study in Iran regarding disrespectful maternity care, the aim of the present study is to determine the prevalence and predictors of perceived disrespectful maternity care among a group of Iranian women.

## Methods

This study is the first phase of a mixed-method study conducted to develop guidelines for improving respectful maternity care [14]. This phase is a cross-sectional study that involved 334 postpartum women who gave birth in public and private hospitals in the city of Tabriz.

### Study’s participants

The study participants were women after vaginal birth who were living in Tabriz city. Exclusion criteria were: a) having a stressful event (divorce, death of a first degree family member, or diagnosis of an incurable or hard-to-cure disease for a family member over the last three months); b) history of depression or any type of mental health disorders that was reported by the participant; c) major neonatal abnormalities; d) mental retardation (by referring to patient’s file and medical history form); and e) deafness.

### Recruitment

Sampling was conducted in the postpartum ward of public (Alzahra, 29 Bahman and Taleghani) and private (Behbood, Nor-e-Nejat, and Shahriyar) hospitals in Tabriz. A total of 334 postpartum women were selected based on the proportion to the number of births in these hospitals three months prior to the study. The first author (KH) assessed all women after birth in each hospital in terms of eligibility until the required sample size from that center was reached. She explained study objectives and methods to eligible women and obtained written informed consent from those willing to participate. Tools included socio-demographic, pregnancy, labour and birth characteristics’ questionnaires and D&A scales, which were completed 6 to 18 h after birth through interviews with participants.

### Data collection tools

Socio-demographic, pregnancy, labour and birth characteristics were collected using a researcher-made questionnaire, whose validity and reliability had been confirmed by ten faculty members of Tabriz University of Medical Sciences. Necessary modifications were made by the research team according to feedback and views expressed by faculty members. The questionnaire included four main categories: a) socio-demographic factors (age, occupation, marriage duration, husband’s education, education level, source of support, and marital satisfaction. The marital satisfaction variable was measured by using a subjective item categorized in three levels including high, moderate and low; b) antenatal factors (planned pregnancy, history of abortion, attendance in prenatal class, place of prenatal care); c) intrapartum factors (place and time of birth, duration of labour, precipitous labour, gestational age, augmentation with oxytocin, birth attendant, hospitalization duration in the labour and delivery room); d) neonatal factors (sex and birth weight, APGAR score at 1 and 5 min, admission to Neonatal Intensive Care Unit (NICU).

Data relating to perceived disrespectful maternity care were collected using a D&A scale, which contains 23 items in seven dimensions: 1. protection of pregnant women against physical harm (6 items); 2. observing women’s rights with regard to being informed about her condition/ informed consent/ choice of childbirth position (8 items); 3. observing women’s privacy and confidentiality (1 item); 4. observing women’s dignity and respect (2 items); 5. receiving equitable care without discrimination (2 items); 6. maintaining care and not neglecting pregnant women (3 items); 7. delayed discharge or detention of pregnant women (1 item). A positive answer to each of the items in one dimension was defined as abuse for that dimension. If a mother was identified as having faced D&A in at least one of the seven dimensions, she was considered “disrespected and abused”. This questionnaire was completed 6 to 18 h after birth and designed by Asefa et al. [15] and approved by MCHIP (Maternal and child health integrated program) [16]. Validity and reliability of this questionnaire have been assessed by the research team in another project and its paper is under review. In our study, Cronbach’s alpha coefficient was obtained as 0.90 and ICC (with 95% CI) as 0.98 (95% CI 0.96 to 0.99).

### Sample size and data analysis

Based on the results of a study in Ethiopia [15] about the prevalence of D&A (*P* = 0.32) and considering d = 0.05, Z = 1.96, and q = 0.68, sample size was determined, needing 334 women.

Quantitative data were analyzed in SPSS-24. Descriptive statistics including frequencies (%) and means (standard deviation) were used to describe women’s characteristics and D&A scale. Relationships of D&A scores with the different characteristics were assessed first using bivariate tests including univariate logistic regression to calculate crude Odds Ratios (cORs). Variables with *p*-values < 0.2 were entered into backward multivariate logistic regression to control for confounding. Results of multivariate logistic regression were presented as adjusted Odds Ratios (aOR) with 95% confidence interval (CI). *P*-value < 0.05 was statistically significant.

## Results

A total of 334 women were included between June and September 2019. Approximately half of them (161; 48.5%) aged between 26 and 35 years. The majority (319; 95.5%) were housewives, with moderate economic status (256; 76.6%). About half of them had high school education (146; 43.7%), and were primiparous women (141; 42.2%). In one out of four women, gestational age was 37 weeks or less (79; 23.7%), and birth weight ranged between 2500 g and 4000 g in (274; 82.0%). Less than half of them (146; 43.7%) were hospitalized less than five hours, and companions in the waiting room were mothers or fathers of pregnant women in nearly half of them (163; 48.8%). Women gave birth during the day (8 a.m. to 8 p.m.) in more than half of cases (192; 57.5%). In more than half of cases (199; 59.6%), the birth attendant were residents or on-call obstetricians. More than three quarters of mothers (264; 79.3%) had episiotomy and oxytocin was used in labour in about half of them (144; 48.3%). Other details are presented in Tables 1 and 2.

**Table 1:** Socio-demographic characteristics among participants (*n* = 334)

Variables	n %
**Age** (Years)	
18–25	132 (39.5)
26–35	161 (48.5)
≥ 36	41 (12.3)
**Work status**	
House keeper	319 (95.5)
Employed	15 (4.5)
**Infertility**	12 (3.6)
**Prenatal class**	67 (20.1)
**Education**	
Elementary and lower	58 (17.4)
Intermediate	79 (23.7)
High school	146 (43.7)
University	51 (15.3)
**Husband’s age** (Years)	
18–25	38 (11.4)
26–35	196 (58.7)
≥ 36	100 (29.9)
**Child birth (Public)**	298 (89.2)
**Economic status**	
Low	24 (7.2)
Moderate	256 (76.6)
High	54 (16.2)
**Marriage duration** (Years)	
≤ 10	278 (83.2)
≥ 11	56 (16.8)
**Marital satisfaction**	214 (64.1)
**Planed pregnancy**	221 (66.2)
**Husband’s education**	
Elementary and lower	65 (19.5)
Intermediate	72 (21.6)
High school	132 (39.5)
University	65 (19.5)
**Husband’s Job**	
Unemployed	12 (3.6)
Employed	20 (6.0)
Self-employed	117 (35.0)
Manual worker	185 (55.4)
**Source of support**	
Husband	27 (8.1)
Mother or father	163 (48.8)
Relative	144 (43.1)
**Place of prenatal care**	
Health center	173 (51.8)
Hospital	21 (6.3)
Private office (Obstetrician)	116 (34.7)
Private office (Midwife)	24 (7.2)
**Gravid**	
One	141 (42.2)
Two	113 (33.8)
Three	55 (16.5)
≥ 4	25 (7.5)
**Abortion history**	
No abortion	259 (77.5)
One	60 (18.0)
≥ 2	15 (4.5)

**Table 2:** Pregnancy, labour and birth characteristics among participants (*n* = 334)

Variables	n %
**Gestational age at birth**	
≤ 37	79 (23.7)
> 37	255 (76.3)
**Prolonged labour (dilatation < 1 cm/h)**	37 (11.1)
**Doula presence**	9 (2.7)
**Birth weight** (Gram)	
≤ 2500	47 (14.1)
2500–4000	274 (82.0)
≥ 4000	13 (3.9)
**Postpartum bleeding (≥ 500 CC)**	25 (7.5)
**Use of labour analgesia**	101 (30.2)
**Augmentation**	144 (48.3)
**Episiotomy**	264 (79.3)
**Hospitalization duration in labour and delivery room (Hour)**	
≤ 5	146 (43.7)
6–10	109 (32.6)
≥ 11	79 (23.6)
**Admission to NICU**	47 (14.1)
**Apgar 1 min**	
≤ 8	12 (3.6)
9–10	322 (96.4)
**Apgar 5 min**	
≤ 8	12 (3.6)
9–10	322 (96.4)
**Macrosomia (Newborn weight ≥ 4000 g)**	13 (3.9)
**Birth attendant**	
Midwife	77 (23.1)
Obstetrician (resident or on-call)	199 (59.6)
Student (midwifery or intern)	16 (4.8)
Personal physician or midwife	42 (12.6)
**Number of health care providers**	
One	42 (12.6)
Two	77 (53.0)
3≤	115 (34.4)
**Giving birth at day** (8 a.m. to 8 p.m.)	192 (57.5)
**Baby sex** (Girl)	161 (48.2)
**Precipitous labour (Labor duration < 3 h)**	17 (5.1)

### Prevalence of D&a

Most women (253; 75.7%) reported one or several types of perceived disrespectful maternity care (Table 3). Receiving no pain relief during labour even when needed, was revealed by more than one-third of women (140; 41.9%). The most frequent type of D&A related to denying women’s rights and preferences, since about half of the women claimed that they were not allowed to choose their position (148; 44.3%) or move during labour (142;42.5%). Women’s privacy was violated in more than one-third of women (112; 35.5%) and they stated that no curtains were used to protect their privacy. Regarding maternal dignity, 54 women (16.2%) reported that staff did not talk to them politely. Women were subjected to discrimination and disrespect in less than one-tenth of different situations (24; 7.2%). Forty-eight women (14.4%) also reported that staff did not encourage them to call if needed. Only three women (0.9%) were detained in hospital against their will (Table 3).

**Table 3:** Prevalence of perceived disrespectful maternity care in terms of total disrespect and abuse (D&A) and its subcategories (*n* = 334)

Categories of D&A	Yes, n % No, n %	Type of D&A	Yes, n %
1, The woman is protected from physical harm or ill treatment.	Yes: 212 (63.5) No: 122 (36.5)	Provider used physical violence (e.g. slapping, beating).	13 (3.9)
		I was physically restrained.	123 (36.8)
		I was deprived of my baby without any medical indication.	4 (1.2)
		I was deprived of food or drinks without any medical permission.	12 (3.6)
		I did not receive any pain-killers, even when desperately needed.	140 (41.9)
		Provider did not provide health care in a culturally appropriate way.	34 (10.2)
2. The woman’s right to information, informed consent, and choice/preference is protected.	Yes: 208 (62.3) No: 126 (37.7)	Provider did not introduce himself/herself to me and my companion.	94 (28.1)
		Provider did not encourage me to ask questions.	71 (21.3)
		provider did not answer my questions promptly and politely	49 (14.7)
		Provider did not explain what was going on and what to expect during birth.	50 (15.0)
		Provider did not periodically inform me about progress of birth.	83 (24.9)
		Provider did not allow me to move during labour.	142 (42.5)
		Provider did not allow me to choose my position during birth.	148 (44.3)
		Provider did not obtain informed consent prior to any action.	94 (28.1)
3. The woman’s confidentiality and privacy is protected.	Yes: 112 (35.5) No: 222 (64.5)	provider did not use curtain or visual barriers to protect privacy	112 (35.5)
4. The woman is treated with dignity and respect.	Yes: 59 (17.7) No: 275 (82.3)	Provider did not talk to me politely**.**	54 (16.2)
		Provider insulted, threatened, intimidated or forced me to do something.	25 (7.5)
5. The woman receives equitable care, free of discrimination.	Yes:30 (9.0) No: 304 (91.0)	Provider spoke in another language or technical jargon that I could not understand.	14 (4.2)
		Provider showed me disrespect in specific ways.	24 (7.2)
6. The woman is left without care/attention	Yes: 64 (19.2) No: 270 (80.8)	Provider did not encourage me to call if needed.	48 (14.4)
		provider did not come immediately, when I called him/her	43 (12.9)
		Provider left me alone or unwatched.	38 (11.4)
7. The woman is detained or confined against her will.	Yes: 3 (0.9) No: 331 (99.10)	I was detained at the health center against my will.	3 (0.9)
**D&A total**	**253 (75.7)**		

### Predictors of perceived disrespectful maternity care

There was a significant correlation between D&A with socio-demographic factors (source of support, marital satisfaction), antenatal factors (place of prenatal care), intrapartum factors (time of birth, type of hospital, birth attendant, hospitalization duration in labour and delivery room, number of healthcare providers, augmentation with oxytocin) (*p* < 0.05) (Tables 4 and 5).

**Table 4:** Correlation between socio-demographic characteristics with perceived disrespectful maternity care among participants (*n* = 334)

Variables	n %	cOR (95%CI)^⁎^
**Age** (Years)		
26–35 (Ref)	121 (47.8)	1
18–25	101 (39.9)	1.07 (0.62 to 1.84)
≥ 36	31 (12.3)	1.02 (0.46 to 2.27)
**Work status**		
House keeper (Ref)	240 (94.9)	1
Employed	13 (5.1)	0.46 (0.10 to 2.11)
**Education**		
High school (Ref)	109 (43.1)	1
Elementary and lower	49 (19.4)	1.84 (0.82 to 4.12)
Intermediate	58 (22.9)	0.93 (0.50 to 1.78)
university	37 (14.6)	0.89 (0.43 to 1.84)
**Marriage duration** (Years)		
≤ 10 (Ref)	216 (85.4)	1
≥ 11	37 (14.6)	0.55 (0.30 to 1.04)
**Marital satisfaction**		
Yes (Ref)	153 (60.5)	1
No	100 (39.5)	1.99 (1.13 to 3.50)^**^
**Husband’s age** (Years)		
26–35 (Ref)	150 (59.3)	1
18–25	30 (11.9)	1.15 (0.49 to 2.68)
≥ 36	73 (28.9)	082 (0.47 to 1.43)
**Economic status**		
Moderate (Ref)	191 (75.5)	1
Low	16 (6.3)	0.68 (0.27 to 1.66)
High	46 (18.2)	1.95 (0.87 to 4.36)
**Husband job**		
Manual worker (Ref)	84 (33.2)	1
Unemployed	9 (3.6)	1.17 (0.30 to 4.62)
Employed	15 (5.9)	1.17 (0.39 to 3.50)
Self-employed	145 (57.3)	1.42 (0.83 to 2.42)
**Husband’s education**		
High school (Ref)	98 (38.7)	1
Elementary and lower	55 (21.7)	1.90 (0.87 to 4.15)
Intermediate	53 (20.9)	0.96 (0.50 to 1.86)
university	47 (18.6)	0.90 (0.46 to 1.76)

*crude Odds Ratio (95% Confidence Interval)

**Significant (*P* < 0.05)

**Table 5:** Correlation between pregnancy, labour and birth characteristics with perceived disrespectful maternity care among postpartum women (*n* = 334)

Variables	n %	cOR (95%CI)^⁎^
**Gravid**		
One (Ref)	112 (44.3)	1
Two	80 (31.6)	0.62 (0.35 to 1.11)
Three	43 (17.0)	0.92 (043 to 1.98)
4≤	18 (7.1)	0.66 (0.25 to 1.74)
**Infertility**		
No (Ref)	243 (96.0)	1
Yes	10 (4.0)	1.62 (0.34 to 7.57)
**Prenatal class attendance**		
No (Ref)	204 (80.6)	1
Yes	49 (19.4)	0.84 (0.45 to 1.54)
**Source of support**		
Mother or father (Ref)	128 (5.6)	1
Husband	15 (5.9)	0.34 (0.14 to 0.79)^**^
relative	110 (43.5)	0.88 (0.51 to 1.51)
**Hospitalization duration in labour and delivery room** (Hour)		
≤ 5 (Ref)	102 (40.3)	1
6–10	85 (33.6)	1.52 (0.86 to 2.71)
≥ 11	66 (26.1)	2.19 (1.09 to 4.37)^**^
**Number of healthcare providers**		
≥ 3 (Ref)	102 (40.3)	1
One	26 (10.3)	0.20 (0.08 to 0.48)^**^
Two	125 (49.4)	0.30 (0.15 to 0.59)^**^
**birth attendant**		
Obstetric (resident or on call) (Ref)	177 (70.0)	1
Midwife	51 (20.2)	0.24 (0.12 to 0.46)^**^
Personal physician or midwife	13 (5.1)	0.05 (0.02 to 0.12)^**^
Student (midwifery or intern)	12 (4.7)	0.37 (0.11 to 1.25)
**Prolonged labour (dilatation < 1 cm/h)**		
No (Ref)	222 (87.7)	1
Yes	31 (12.3)	1.74 (0.70 to 4.34)
**Child birth (public)**		
Yes (Ref)	244 (96.4)	1
No	9 (3.6)	0.07 (0.03 to 0.16)^**^
**Postpartum bleeding** (≥ 500 CC)		
No (Ref)	231 (91.3)	1
Yes	22 (8.7)	2.46 (0.72 to 8.49)
**Precipitous labour (childbirth < 3 h)**		
No (Ref)	243 (96.0)	1
Yes	10 (4.0)	0.05 (0.15 to 0.26)^**^
**Baby sex**		
Boy (Ref)	125 (49.4)	1
Girl	128 (50.6)	1.48 (0.89 to 2.47)
**Place of prenatal care**		
Health center (Ref)	144 (56.9)	1
Private office (Obstetrician)	16 (6.3)	0.32 (0.19 to 0.59)^**^
Private office (Midwife)	73 (28.9)	0.40 (0.15 to 1.02)
Hospital	20 (7.9)	4.02 (0.52 to 31.21)
**Planed pregnancy**		
Yes (Ref)	169 (66.8)	1
No	84 (33.2)	1.83 (1.03 to 3.25)^**^
**Abortion history**		
No abortion(Ref)	194 (76.7)	1
One	47 (18.6)	1.21 (0.61 to 2.38)
≥ 2	12 (4.7)	1.34 (0.36 to 4.89)
**Doula presence**		
No (Ref)	248 (98.1)	1
Yes	5 (2.0)	0.38 (0.10 to 1.48)
**Birth weight (Gram)**		
2500–4000	34 (13.4)	1
2500≥	211 (83.4)	1.52 (0.86 to 2.71)
4000≤	8 (3.2)	2.19 (1.09 to 4.37)**
**Gestational age at birth**		
> 37 (Ref))	193 (76.3)	1
≤ 37	60 (23.7)	1.01 (0.56 to 1.83)
**Apgar 1 min**		
9–10 (Ref)	243 (96.0)	1
≤ 8	10 (4.0)	1.62 (0.34 to 7.57)
**Apgar 5 min**		
9–10 (Ref)	243 (96.0)	1
≤ 8	10 (4.0)	0.61 (0.13 to 2.86)
**use of labour analgesia**		
No (Ref)	182 (71.9)	1
Yes	71 (28.1)	0.66 (0.39 to 1.12)
**Time of giving birth**		
Day (8 a.m. to 8 p.m.) (Ref)	129 (51.7)	1
Night	124 (49.0)	3.36 (1.88 to 6.02)**
**Augmentation**		
No (Ref)	133 (52.6)	1
Yes	120 (47.4)	1.70 (1.01 to 2.87)**
**Admission to NICU**		
No (Ref)	215 (85.0)	1
Yes	38 (15.0)	1.41 (0.65 to 3.6)

* Crude Odds Ratio (95% Confidence Interval)

**Significant (*P* < 0.05)

Variables of marriage duration, marital satisfaction, place of prenatal care, hospitalization duration in labour, delivery room, number of healthcare providers, use of labour analgesia, postpartum bleeding, augmentation and baby sex were removed from the model.

Multivariate logistic regression showed that nighttime childbirth (aOR 3.07; 95% CI 1.61 to 5.88) increased the likelihood of perceived disrespectful maternity care. However, presence of spouses to accompany their wives in waiting rooms (aOR 0.32; 95% CI 0.11 to 0.88), the attendance of private physicians or midwives (aOR 0.05; 95% CI 0.02 to 0.12), and midwives (aOR 0.22; 95% CI 0.11 to 0.45) decreased the likelihood of perceived disrespectful maternity care (Table 6).

**Table 6:** Multivariable Regression Logistic model for perceived disrespectful maternity care and influencing factors (n = 334)

Variables	aOR (95%CI)^⁎^	cOR (95%CI)^+^
**Time of giving birth**		
Day (8 a.m. to 8 p.m.)	1	1
Night (Ref)	3.07 (1.61 to 5.88)^**^	3.36 (1.88 to 6.02)^**^
**Source of support**		
Mother or father (Ref)	1	1
Husband	0.32 (0.11 to 0.88)^**^	0.34 (0.14 to 0.79)^**^
Relative	1.26 (0.68 to 2.36)	0.88 (0.51 to 1.51)
**Birth attendant**		
Obstetric (resident or on call)(Ref)	1	1
Midwife	0.22 (0.11 to 0.45)^**^	0.24 (0.12 to 0.46)^**^
Personal physician or midwife	0.05 (0.02 to 0.12)^**^	0.05 (0.02 to 0.12)^**^
Student (midwifery or intern)	0.35 (0.10 to 1.24)	0.37 (0.11 to 1.25)

*adjusted Odds Ratio (95% Confidence Interval) ^+^ Crude Odds Ratio (95% Confidence Interval) **Significant (*P* < 0.05)

Adjusted for all other socio-demographic variables with P < 0.2 in the bivariate analysis. Variables of marriage duration and marital satisfaction were removed from the model

Adjusted for all other pregnancy variables with *P* < 0.2 in the bivariate analysis. Variable of place of prenatal care was removed from the model

Adjusted for all labour and childbirth variables with P < 0.2 in the bivariate analysis. Variables of hospitalization duration in labour and delivery room, number of healthcare providers, use of labour analgesia, postpartum bleeding, augmentation and baby sex were removed from the model

## Discussion

In our study, three out of every four women reported perceived disrespectful maternity care, less frequent than in Nigeria (98%) and Ethiopia (78%), but higher than in Kenya (20%), Tanzania (15%), India (57%) and European countries (20%) [8, 9, 11, 15, 17, 18]. More respectful maternity care was significantly related with daytime delivery, delivery by private obstetricians or private midwives or midwives, and presence of spouses in the waiting room.

High levels of perceived disrespectful maternity care in Iran may appear to be due to the fact that childbirth in Iran is not women-centered and mainly focused on medical interventions. Episiotomy for primiparous women and use of oxytocin during labour are regarded as acceptable and routine procedures in Iran without informed consent of women or any involvement in the decision making process [19, 20]. Whereas in European countries less than half of the women receive oxytocin or episiotomy during labour [21, 22] and routine use of oxytocin or episiotomy without permission of the woman is considered obstetric violence [23, 24].

The most frequent types of perceived disrespectful maternity care related to not allowing women to choose labour position, limitations in mobility, not using analgesics, and not using a curtain or partition to maintain privacy. In a study in Ethiopia (2015), the most frequent type of D&A was disregard for women’s choices and preferences, which is in agreement with our findings [15]. An important cause of perceived disrespectful maternity care was no pain relief during labour. Another study in Iran showed that fear of labour pain was the reason for 40% of cesarean sections [25].

The presence of spouses to accompany their wives reduced possible perceived disrespectful maternity care by up to 68%. Several studies in low and middle income countries including Iran [26–30] analyzed benefit and problems of men participation to childbirth. Most studies concluding for prevalence of benefit [26–30] and a minority reported cultural, personal, interpersonal, health system-related barriers or socio-economic barriers [28].

Births guided by obstetric residents increased perceived disrespectful maternity care compared to guidance by private physicians or midwives. This high level of perceived disrespectful maternity care can be attributed to the small numbers of obstetric residents in Iran, their huge workload in public hospitals, their fatigue due to 36-h shifts and their consequent sleeplessness. A review from Iran showed high rates of burnout among obstetric residents [31]. Evidence showed that training residents may be associated with occupational burnout that it can lead to reduced patient satisfaction with treatment [32]. This is in agreement with Segrin & Passalacqua (2012) reporting about residents’ high level of stress having a significant relationship with increased occupational burnout and reduced empathy with patients [33]. Empathy and patient-centered relationships were lower in prolonged shifts, particularly in the second half of the shift [33].

Nighttime childbirth increased the likelihood of perceived disrespectful maternity care. Since the number of healthcare providers and physicians in the night shift is low and also there is no physician (physicians are resident or on call) or head nurse to coordinate matters, night shift staff are also responsible for the affairs of the ward in addition to providing care for parturient women which can also cause tiredness, sleepiness and increased responsibility in night shift staff. As such, the result of one study have shown that sleepiness and tiredness caused by night shift can adversely affect the staff’s medical decisions, memory, mood and behavior, including aggression, anxiety, and depression, and ultimately patient safety [34].

### Strengths and limitations

Inclusion of multi- and primiparous women with term and preterm births as well as singleton and twin births can be regarded as strong points of our study, which also included sampling from public and private hospitals. Possibility of recall bias was low since questionnaires were completed immediately after childbirth. However, since questionnaires were completed in the hospital setting, mothers may have under-reported D&A for fear of not being able to use medical services after childbirth. Therefore it is recommended that future follow-up studies assess perceived disrespectful maternity care in their own homes. To reduce this limitation, all mothers had been assured that this study would not affect receiving future care by any means. Main limitation of the study is that the perception of mothers from disrespectful maternity care was measured subjectively due to the difficulties in producing objective measurement of it. Perception may also determine overestimation of the phenomenon. Mothers may perceive some D&A behaviors as normal, and underreport such behaviors. Therefore, it is suggested that mothers be taught RMC principles and be familiarized with their rights in childbirth preparation classes. Another major weakness of this study is that dichotomization of such a complex outcome (D&A) does not permit to stratify the magnitude of the problem. A positive answer to each of the items in one dimension was defined as abuse for that dimension. Another limitation included sampling from Tabriz city only, where all mothers used Azari language. Therefore it is recommended to conduct this study also in other parts of Iran with different cultures and ethnicities.

## Conclusions

This study showed high levels of perceived disrespectful maternity care in a group of postpartum Iranian women, which could be a serious warning to policy-makers to promote respectful and less-interventionist childbirth. Labor is a major stressor both for patients but also for operators and this is confirmed by increased D&A during nightshifts. Low resources settings need to have a tradeoff between optimization use of human resources and optimal care for patients. This paper highlights the importance of quantifying the magnitude of D&A in order to intervene in that component which is easily preventable. Other studies in similar setting have also suggested interventions such as client awareness-building regarding patient rights and focusing on the importance of empathy to reduce disrespectful maternity care [35–37]. These interventions can be at different levels: a) policy and governance (ethic guidelines); b) health system (provider attitude); c) community (individual attitude) [37]. It is recommended that the presence of spouses during childbirth is encouraged through participation in “easy childbirth” classes. Other interventions to be implemented include increasing the numbers of night shift staff; reducing the numbers of working shifts at night and training obstetric residents and midwives on ethical considerations by holding ethics classes, with particular emphasis on empathy with patients about women’s rights and their preferences regardless of their socio-economic background.

## Data Availability

The datasets used and analyzed during the current study are available from the corresponding author on reasonable request.

## Abbreviations

D&A: Disrespect and Abuse
RMC: Respectful Maternity Care
ICC: Intra-class Correlation Coefficient
CI: Confidence Interval
OR: Odds Ratio
ICPD: International Conference on Population and Development
NICU: Neonatal Intensive Care Unit
WHO: World Health Organization
